# Serotonin‐deficient neonatal mice are not protected against the development of experimental bronchopulmonary dysplasia or pulmonary hypertension

**DOI:** 10.14814/phy2.15482

**Published:** 2022-10-06

**Authors:** Danielle S. Roberts, Laura G. Sherlock, Janelle N. Posey, Jamie L. Archambault, Eva S. Nozik, Cassidy A. Delaney

**Affiliations:** ^1^ Section of Neonatology, Department of Pediatrics University of Colorado Anschutz Medical Campus Aurora Colorado USA; ^2^ Department of Pediatrics University of Colorado Anschutz Medical Campus Aurora Colorado USA; ^3^ Pediatric Critical Care Medicine, Department of Pediatrics University of Colorado Anschutz Medical Campus Aurora Colorado USA; ^4^ Cardiovascular Pulmonary Research Laboratories Aurora Colorado USA

**Keywords:** BPD, neonate, pulmonary hypertension, serotonin

## Abstract

Serotonin (5‐hydroxytryptamine, 5‐HT) is a potent pulmonary vasoconstrictor and contributes to high pulmonary vascular resistance in the developing ovine lung. In experimental pulmonary hypertension (PH), pulmonary expression of tryptophan hydroxylase‐1 (TPH1), the rate limiting enzyme in 5‐HT synthesis, and plasma 5‐HT are increased. 5‐HT blockade increases pulmonary blood flow and prevents pulmonary vascular remodeling and PH in neonatal models of PH with bronchopulmonary dysplasia (BPD). We hypothesized that neonatal *tph1* knock‐out (KO) mice would be protected from hypoxia‐induced alveolar simplification, decreased vessel density, and PH. Newborn wild‐type (WT) and *tph1* KO mice were exposed to normoxia or hypoxia for 2 weeks. Normoxic WT and KO mice exhibited similar alveolar development, pulmonary vascular density, right ventricular systolic pressures (RVSPs), and right heart size. Circulating (plasma and platelet) 5‐HT decreased in both hypoxia‐exposed WT and KO mice. *Tph1* KO mice were not protected from hypoxia‐induced alveolar simplification, decreased pulmonary vascular density, or right ventricular hypertrophy, but displayed attenuation to hypoxia‐induced RVSP elevation compared with WT mice. *Tph1* KO neonatal mice are not protected against hypoxia‐induced alveolar simplification, reduction in pulmonary vessel density, or RVH. While genetic and pharmacologic inhibition of tph1 has protective effects in adult models of PH, our results suggest that tph1 inhibition would not be beneficial in neonates with PH associated with BPD.

## Graphical abstract

Serotonin‐deficient neonatal mice are not protected against hypoxia‐induced alveolar simplification, reduction in pulmonary vessel density, or RVH. While genetic and pharmacologic inhibition of tryptophan hydroxylase 1 (TPH1) has protective effects in adult models of pulmonary hypertension (PH), our results suggest that tph1 inhibition would not be beneficial in neonates with PH associated with BPD.

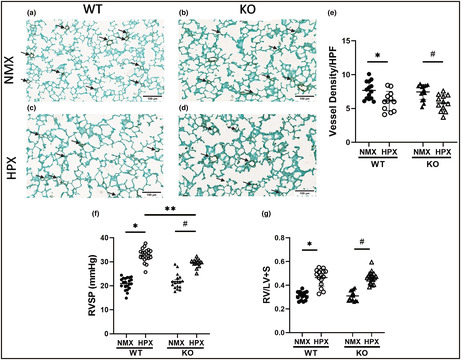

## 1 INTRODUCTION

Bronchopulmonary dysplasia (BPD) is a disorder of prematurity that results in potential lifelong respiratory morbidity (Abman et al., 1985; Northway et al., 1967). The incidence of BPD is rising, despite both technological and clinical advances in neonatal care (Stoll et al., 2015). Fourteen to 25% of infants with BPD develop pulmonary hypertension (PH; An et al., 2010; Bhat et al., 2012; Mourani et al., 2015). While most therapies for PH associated with BPD are targeted at NO/cGMP signaling, alterations in numerous pathways, including serotonin (5‐hydroxytryptamine [5‐HT]), have been implicated in the development of PH associated with BPD (Bhatt et al., 2001; Delaney et al., 2018; Le Cras et al., 2002; Mourani et al., 2004, 2009). 5‐HT may contribute to PH, by several mechanisms including pulmonary vasoconstriction, pulmonary artery smooth muscle cell proliferation, and activation of pulmonary fibroblasts leading to fibrosis (Chen et al., 2014; Delaney et al., 2013; Lawrie et al., 2005; MacLean et al., 1996; Morecroft et al., 1999). Furthering our understanding of the underlying mechanisms surrounding BPD associated with PH is essential in the prevention and treatment of this prevalent disease.

5‐Hydroxytryptamine has largely been studied for its role in the central nervous system, where it regulates mood, memory, and pain. However, the majority of 5‐HT is synthesized in the periphery by enterochromaffin cells of the small intestine where it has diverse biologic roles acting as a mitogen, growth factor, regulator of vasomotor tone, and mediator of inflammation (Berger et al., 2009; Fanburg & Lee, 1997; MacLean et al., 1996; Watts, 1996; Watts et al., 1994, 2012). The ability of 5‐HT to influence such a wide variety of functions is attributed to its varied receptor system and intricate signaling pathways (Adnot et al., 2013). 5‐HT is synthesized from L‐tryptophan through the activity of tryptophan hydroxylase (TPH). TPH is the rate limiting step in 5‐HT synthesis and exists as two isoforms, *tph1* and *tph2*, encoded by separate genes (Walther et al., 2003). While *tph1* expression is highest in enterochromaffin cells of the small intestine, other cell types, including pulmonary artery endothelial cells, express *tph1* (Barter & Pearse, 1953; Delaney et al., 2018; Gershon et al., 1977; Walther et al., 2003). *Tph2* expression is limited to the brain and enteric nervous system. As *tph2* is expressed in the enteric nervous system, *tph1* knock‐out (KO) mice synthesize peripheral 5‐HT via tph2 (Gershon et al., 1977; Walther et al., 2003). Plasma levels of 5‐HT are in the low nanomolar range as circulating 5‐HT is rapidly taken up by platelets via the 5‐HT transporter (SERT) and stored in platelet dense granules (Da Prada & Picotti, 1979; Holmsen & Weiss, 1979).

The “serotonin hypothesis of PH” was developed in the 1960s after the development of PH was observed in patients consuming diet pills that increase 5‐HT bioavailability (Eddahibi & Adnot, 2002; MacLean, 2018). Increasing clinical and experimental evidence support a role for aberrant 5‐HT signaling in the pathogenesis of neonatal PH and BPD. 5‐HT immunoreactive cells are present from 8 weeks gestation onward in human fetal lungs and infants who died from severe BPD exhibit a 34‐fold increase in pulmonary 5‐HT when compared with age‐matched controls (Cutz et al., 1985; Johnson et al., 1985). Maternal selective 5‐HT reuptake inhibitor (SSRI) use in the third trimester of gestation is associated with a sixfold increased risk of persistent pulmonary hypertension of the newborn (PPHN; Chambers et al., 2006). In addition, absent SERT expression in the neonatal lung is associated with alveolar capillary dysplasia, a fatal disease characterized by PH (Castro et al., 2017). Newborn rats exposed to an SSRI, fluoxetine, in utero develop pulmonary vascular remodeling, abnormal oxygenation, and higher mortality when compared with controls (Belik, 2008).

Our lab has demonstrated that 5‐HT is a potent pulmonary vasoconstrictor in the ovine fetus and that 5‐HT contributes to fetal pulmonary vascular resistance (Delaney et al., 2011). Pulmonary TPH1 expression and pulmonary artery endothelial cell (PA‐EC) synthesis of 5‐HT is increased in fetal sheep with PH (Delaney et al., 2013). Our lab has also demonstrated that both lung TPH1 expression and plasma 5‐HT are increased in neonatal mice with bleomycin‐induced PH and BPD (Delaney et al., 2018). Additionally, we have shown increased activation and accumulation of platelets, the primary source of circulating 5‐HT, in the lungs of mice with bleomycin‐induced neonatal PH (Davizon‐Castillo et al., 2020).

Due to increasing support regarding the role of 5‐HT in promoting PH, genetic or pharmacologic depletion of 5‐HT has been the focus of multiple studies. Overall, the results demonstrate that 5‐HT depletion affords varying degrees of protection in several experimental adult rodent models of PH (Abid et al., 2012; Aiello et al., 2017; Izikki et al., 2007; Morecroft et al., 2007). From a neonatal perspective, pharmacologic blockade of 5‐HT via inhibition of the 2A receptor not only decreases fetal pulmonary vascular resistance in the ovine fetus with PH, but also protects against the development of PH and pulmonary vascular remodeling in a neonatal murine model of bleomycin‐induced BPD and PH (Delaney et al., 2013, 2018). In the present study, we hypothesized that circulating (platelet and plasma) and pulmonary 5‐HT is increased in neonatal WT hypoxic mice and *tph1* KO neonatal mice would be protected against hypoxia‐induced alveolar simplification, decreased pulmonary vessel density, and PH. To study our hypothesis, we utilized a hypobaric hypoxia neonatal murine model of PH and BPD.

## 2 METHODS

### 2.1 Mouse model

The University of Colorado Denver Institutional Animal Care and Use Committee (IACUC) approved all animal studies. C57BL/6 wild‐type (WT) mice were purchased from Jackson Laboratory and bred in Denver. *Tph1* KO mice were engineered by Dr. M. Bader (Max‐Delbrück‐Center for Molecular Medicine) on a C57BL/6 background (Deruelle et al., 2006). These mice were kindly donated to our laboratory and bred in Denver. Offspring were obtained from crosses of WT mice, *tph1* heterozygous mice, and *tph1* KO mice. Offspring were either raised at Denver altitude (633 mmHg) or placed in hypobaric chambers 24–36 h after birth at 446 mmHg to simulate 12% FiO_2_ at sea level. This murine injury model of PH and BPD produces similar major pathologic findings to infants with PH and BPD including impaired alveolar development (decreased surface area [SA], increased mean linear intercept), vascular injury (decreased vessel density), and pulmonary hypertension (elevated right ventricular systolic pressure and right ventricular hypertrophy; Balasubramaniam et al., 2003; Deruelle et al., 2006; Tang et al., 2000). Hemodynamic assessment and euthanization for blood and tissue collection took place at 2 weeks of age.

### 2.2 Preparation of mouse blood and measurement of platelet and plasma 5‐HT by ELISA

Mice were anesthetized with 1%–2% isoflurane and blood was obtained via cardiac puncture of the right ventricle (RV) after performing a bilateral thoracotomy using a 21‐gauge needle containing heparin. Platelet‐rich plasma (PRP) was obtained by centrifugation of whole blood at 100 *g* for 10 min. PRP was supplemented with prostacyclin (PGI2; 1 μg/mL) and incubated at room temperature for 3 min prior to centrifugation at 2000 *g* for 2 min to obtain platelet poor plasma (PPP) or platelet pellets. 5‐HT levels were measured using the mouse 5‐HT ELISA kit (GenWay Biotech) following the manufacturer's instructions.

### 2.3 Preparation of mouse lung homogenates and measurement of lung 5‐HT by ELISA

Flushed lungs were obtained from storage at −80°C and placed on ice. The reagents, Pierce's Tissue Protein Extraction Reagent, Sigma's Phosphatase Inhibitor Cocktail 2, Sigma's Phosphatase Inhibitor Cocktail 3, and Sigma's Protease Inhibitor Cocktail were prepared per manufacturer's instructions. Tissue samples (25–30 mg) were separated and placed in the Bead Ruptor_12_ (Omni International), run at High Speed for 45 s, and then transferred to ice ×3 cycles. Homogenized samples were incubated for 30 min on ice, then centrifuged at 10,000 *g* for 5 min. 5‐HT levels were measured using the 5‐HT ELISA kit (ALPCO) following the manufacturer's instructions.

### 2.4 Immunohistochemistry

Lungs were inflation‐fixed at 25 cm H_2_O for 30 min with 4% paraformaldehyde for paraffin embedding. Lung sections were stained with hematoxylin and eosin to assess alveolar structure, and rabbit anti‐vWF (1:1500; Sigma‐Aldrich), and ready‐to‐use horse radish peroxidase (HRP) conjugated anti‐rabbit IgG (Dako EnVision + Dual Link System‐HRP [DAB+]) to assess vessel density. von Willebrand Factor (vWF)‐stained slides were developed with Vector very intense purple peroxidase (HRP) substrate kit (Vector Laboratories) and counterstained with light green counterstain.

### 2.5 Evaluation of alveolar development and pulmonary vessel density

Surface area (mm^2^/HPF) and mean linear intercept (MLI) were obtained with Metamorphic Basic (Molecular Devices Sunnyvale). Ten randomly selected non‐overlapping sections per mouse at 20× magnification were assessed. Fields with large airways or vessels were excluded. Vessel density was assessed by counting the number of vessels <30 μm staining positive for vWF per high‐power field (20×). Again, lung fields containing large vessels or airways were excluded, and greater than six fields were included per mouse. An investigator blinded to the experimental group performed the analysis.

### 2.6 Hemodynamic measurements and evaluation of right ventricular hypertrophy

At 2 weeks of life, mice underwent direct RV puncture via closed chest to obtain right ventricular systolic pressures (RVSPs). Hearts were removed and dissected to isolate the free wall of the RV from the left ventricle (LV) and septum (S). Fulton's index, the ratio of RV weight over LV + S weight (RV/LV + S), was used as an index of RV hypertrophy resulting from PH, as previously described (Delaney et al., 2018). An investigator blinded to the experimental group performed the analysis.

### 2.7 RNA isolation and qPCR

RNA was isolated as previously described using a RNeasy kit (Qiagen); then cDNA was prepared using the iScript cDNA synthesis kit (Bio‐Rad; Good et al., 2018). qPCR was performed on a QuantStudio 6 Flex qPCR machine (Thermo Fisher Scientific) using TaqMan Fast Advanced Master Mix (Thermo Fisher Scientific). The following Taqman probe was used: *tph1* (Mm01202614_m1), *tph2* (Mm00557722_m1). All samples were run in triplicate fashion, analyzed, and presented by 2^−ΔΔ*Ct*^ method.

### 2.8 Statistical analysis

Data were analyzed using Prism (GraphPad Software) by one‐way ANOVA, two‐way ANOVA, and unpaired *t*‐test when appropriate. Post hoc analysis was performed using Tukey's posttest when significant differences existed between groups. Data were expressed as mean ± SD and significance defined as *p* < 0.05.

## 3 RESULTS

### 3.1 Circulating and lung 5‐HT is decreased in *tph1* KO mice

**FIGURE 1:**
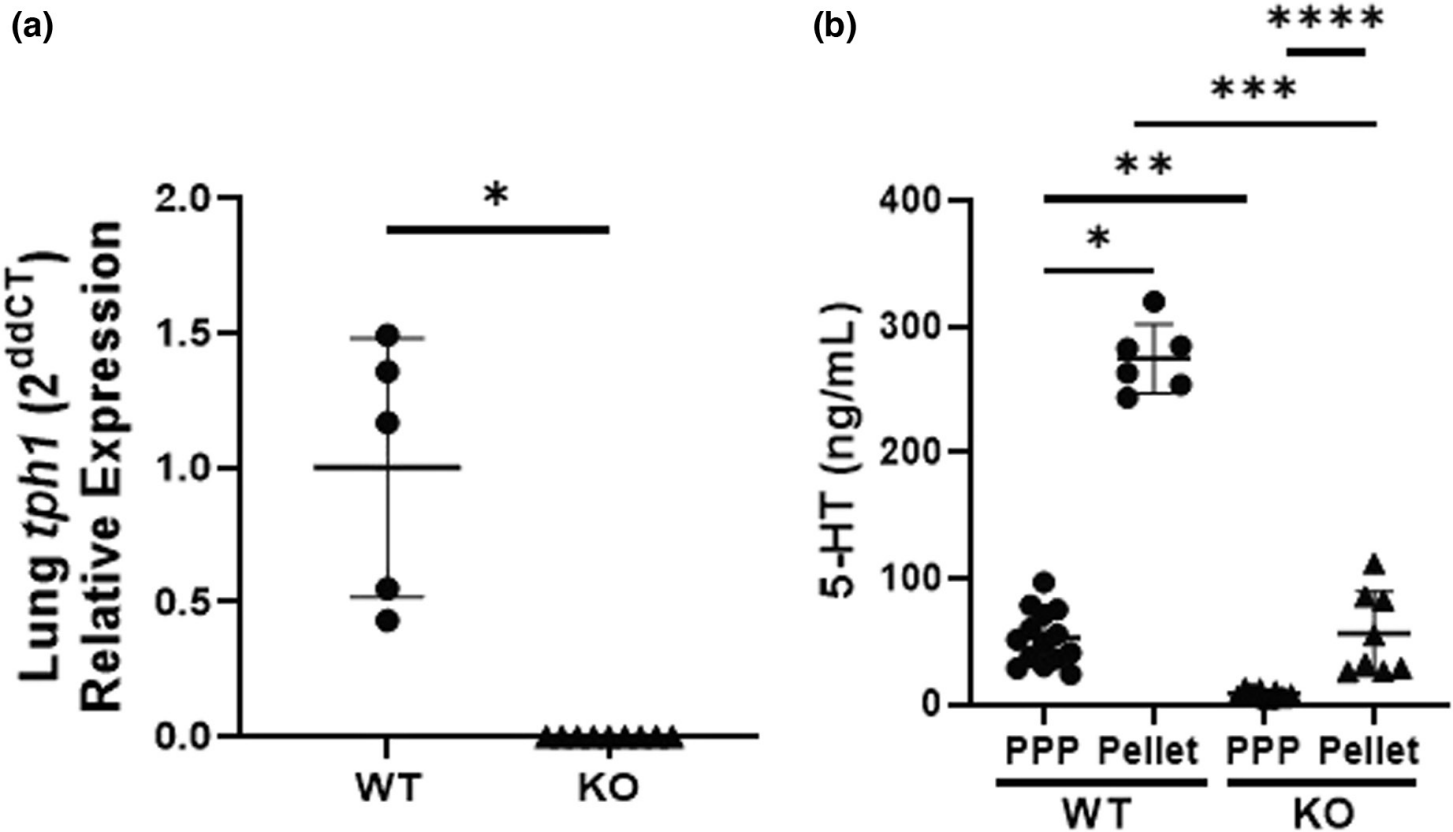
PPP and platelet 5‐HT (ng/ml) are decreased in 2‐week‐old *tph1* KO mice compared with WT mice. (a) Lung *tph1* gene expression in WT and *tph1* KO mice, *p < 0.0001 by unpaired test, *n* = 5 WT (3M, 2F), *n* = 9 KO (5M, 4F), ns for sex. (b) Baseline 5‐HT (ng/ml) in PPP and platelet pellets of WT and *tph1* KO mice, *p < 0.0001, **p < 0.01, ***p < 0.0001, ****p < 0.005 by one‐way ANOVA, *n* = 20 WT (12M, 8F), *n* = 16 KO (9M, 7F), ns for sex. 5‐Ht, 5‐hydroxytryptamine; PPP, platelet poor plasma; WT, wild‐type.

We evaluated our model by first quantifying pulmonary *tph1* gene expression and circulating 5‐HT in WT and *tph1* KO mice. Pulmonary expression of the *tph1* gene was not detected in *tph1* KO mice (Figure 1a). As expected, we found that 5‐HT is primarily located within platelets and 5‐HT levels in *tph1* KO mice are decreased in both platelet poor plasma (PPP) and platelets compared with WT mice (Figure 1b, *p < 0.0001, **p < 0.01, ***p < 0.0001, ****p < 0.005). Although greatly decreased, *tph1* KO mice synthesize 5‐HT in the periphery. The average plasma 5‐HT level in *tph1* KO mice is ~6 ng/ml, while the average platelet 5‐HT level in *tph1* KO mice is ~30 ng/ml, compared with average 5‐HT levels of ~30 and ~285 ng/ml, respectively, in WT mice (Figure 1b).

### 3.2 Two‐week‐old *tph1* KO normoxic mice exhibit similar alveolar development, pulmonary vessel density, pulmonary pressures, and right heart size to WT normoxic mice

**FIGURE 2:**
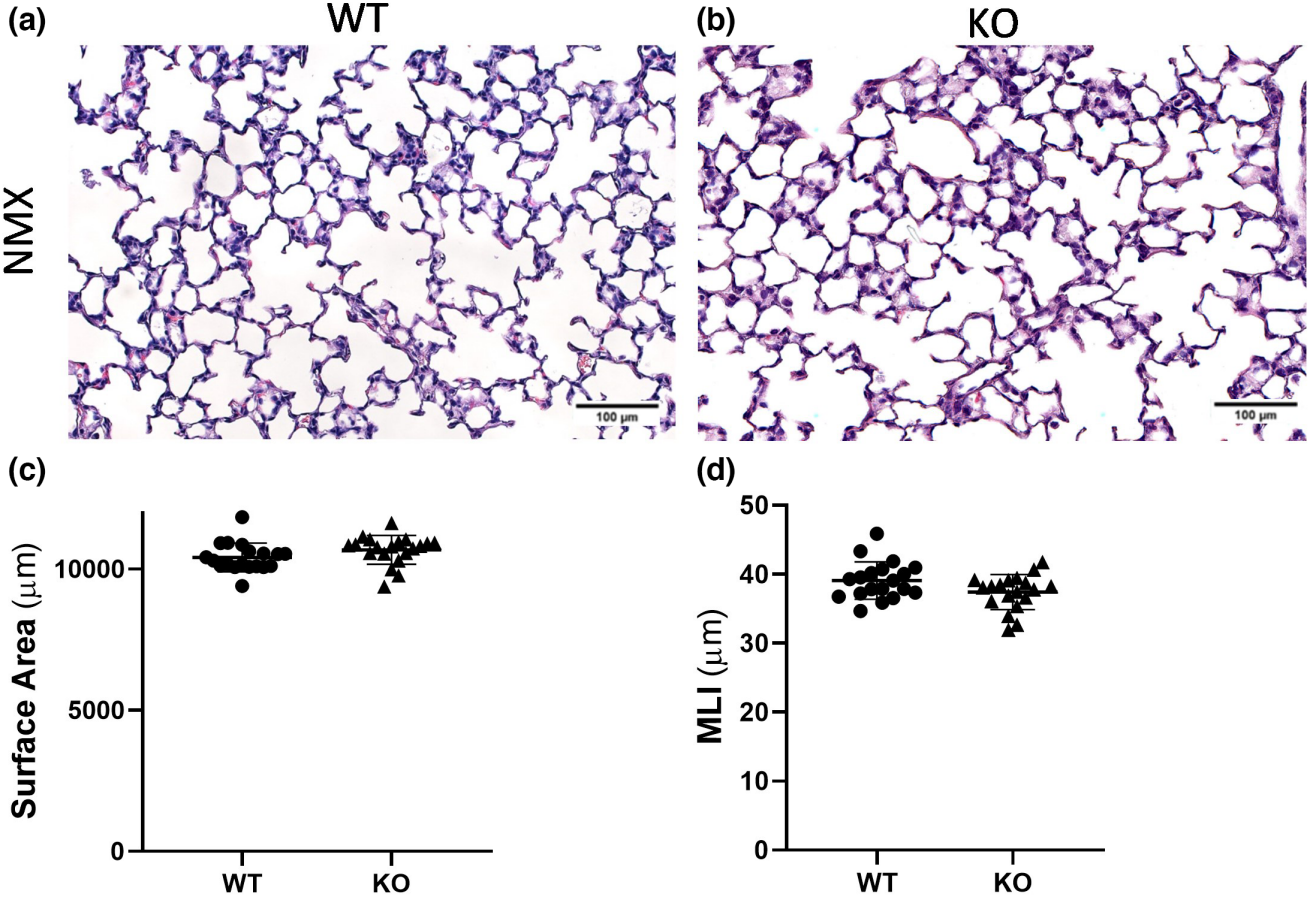
Two‐week‐old *tph1* KO mice exhibit similar alveolar development to WT mice at baseline. (a, b) Representative images of hematoxylin and eosin stained lung sections from 2‐week‐old WT and *tph1* KO mice under control conditions. (c, d) Morphometric analysis, SA, and MLI of WT and *tph1* KO mice under control conditions, ns for genotype and sex by unpaired *t*‐test, *n* = 19 WT (12M, 7F), *n* = 20 KO (11M, 9F), ns for sex, for both MLI and SA. KO, knock‐out; MLI, mean linear intercept; NMX, normoxia; SA, surface area; WT, wild‐type.

**FIGURE 3:**
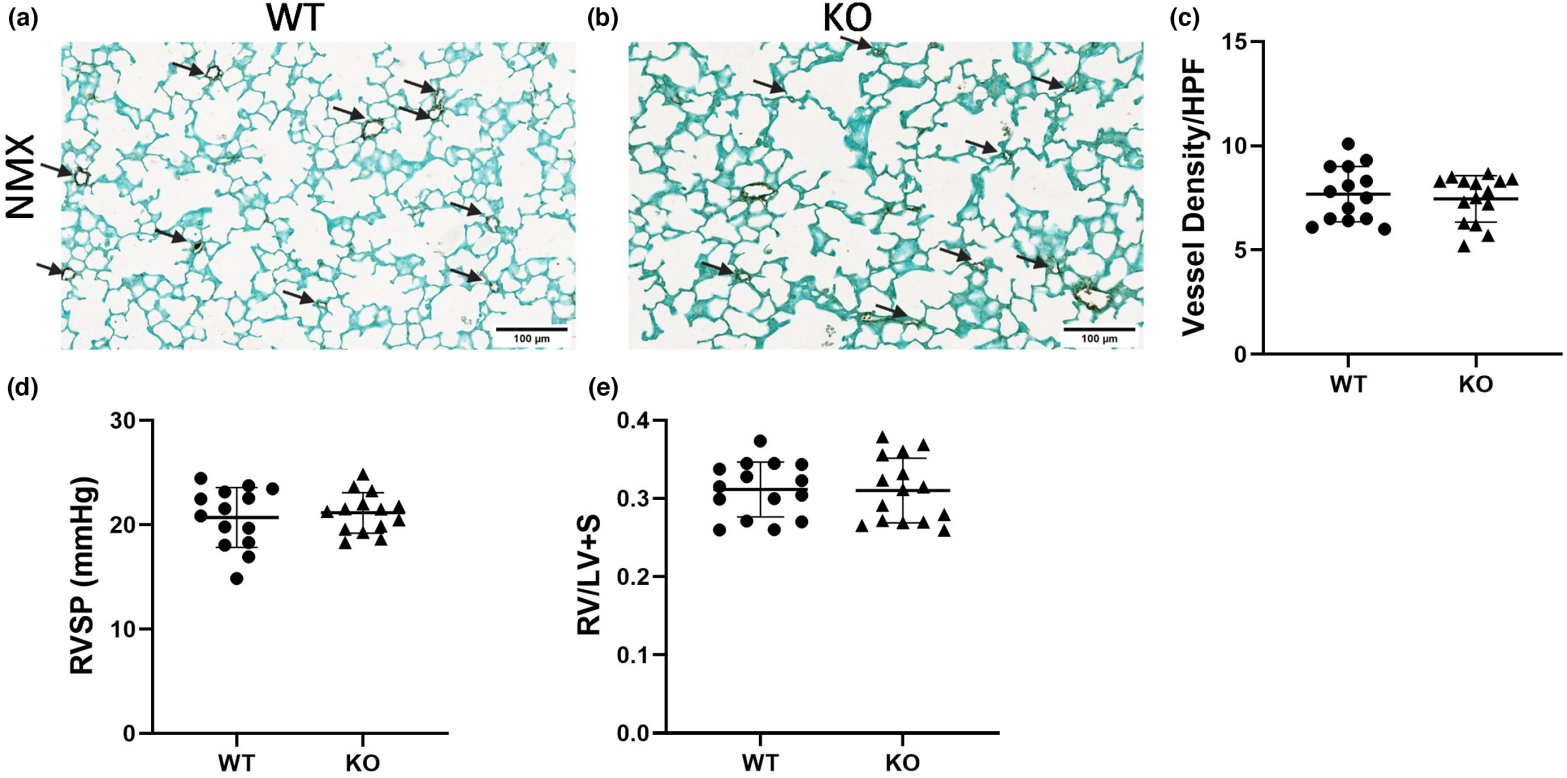
Vessel density, pulmonary artery pressures, and right heart size is similar at 2 weeks of age between WT and KO mice at baseline. (a, b) Representative images of von Willebrand Factor (vWF; purple) staining in WT and *tph1* KO mice in baseline conditions. Arrows indicate vessels <30 μm, scale bar = 100 μm. (c) The number of small vessels <30 μm in WT and *tph1* KO mice at baseline, ns for genotype and sex by unpaired *t*‐test, *n* = 14 WT (8M, 7F), *n* = 15 KO (7M, 8F). (d) Right ventricular systolic pressure (RVSP) in WT and *tph1* KO mice at baseline, ns for genotype and sex by unpaired *t*‐test, *n* = 14 (9M, 5F) for both WT and KO mice. (e) RV/LV + S weights in WT and *tph1* KO mice at baseline, ns by unpaired *t*‐test, *n* = 15 (9M, 6F) for both WT and KO mice. KO, knock‐out; NMX, normoxia, RV/LV + S, right ventricle divided by left ventricle plus septum; WT, wild‐type.

We evaluated alveolar development by examining alveolar SA and MLI. We quantified the density of vWF stained vessels <30 μm. Alveolar development (Figure 2a–d) and pulmonary vessel density (Figure 3a–c) are not altered at 2 weeks of age in *tph1* KO mice compared with WT mice at baseline. Additionally, there is no difference in RVSP or RVH in *tph1* KO mice compared with WT mice at baseline (Figure 3d–e).

### 3.3 *Tph1* KO mice display comparable hypoxia‐induced alveolar simplification and reduction in pulmonary vessel density to WT mice

**FIGURE 4:**
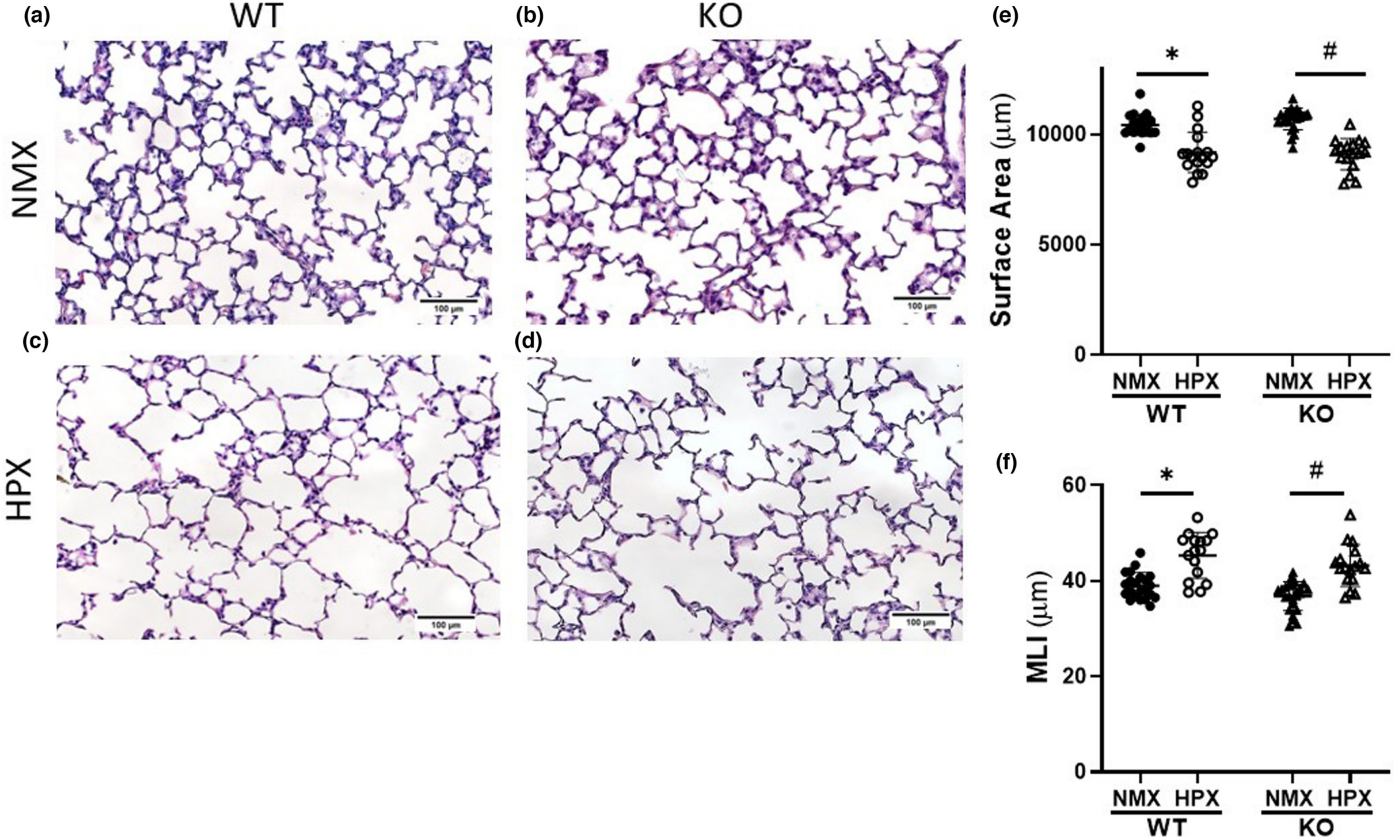
*tph1* KO mice display comparable hypoxia‐induced alveolar simplification compared with WT mice. (a–d) Representative images of hematoxylin and eosin stained lung sections from 2‐week‐old WT and *tph1* KO mice in normoxia and hypoxia, scale bar = 100 μm., (e) SA of WT and TPH1 KO mice in NMX and HPX, *p < 0.0001 and ^#^ *p* < 0.0001 by two‐way ANOVA, *n* = 22 WT NMX (13M, 9F), *n* = 24 KO NMX (12M, 12F), *n* = 17 WT and KO HPX (8M, 9F), ns for sex. (f) MLI of WT and *tph1* KO mice in NMX and HPX, *p < 0.0001 and ^#^ *p* < 0.0001 by two‐way ANOVA, *n* = 22 WT NMX (13M, 9F), *n* = 24 KO NMX (12M, 12F), *n* = 17 WT HPX (8M, 9F), *n* = 18 KO HPX (10M, 8F), ns for sex. HPX, hypoxia; KO, knock‐out; MLI, mean linear intercept; NMX, normoxia; SA, surface area; WT, wild‐type.

**FIGURE 5:**
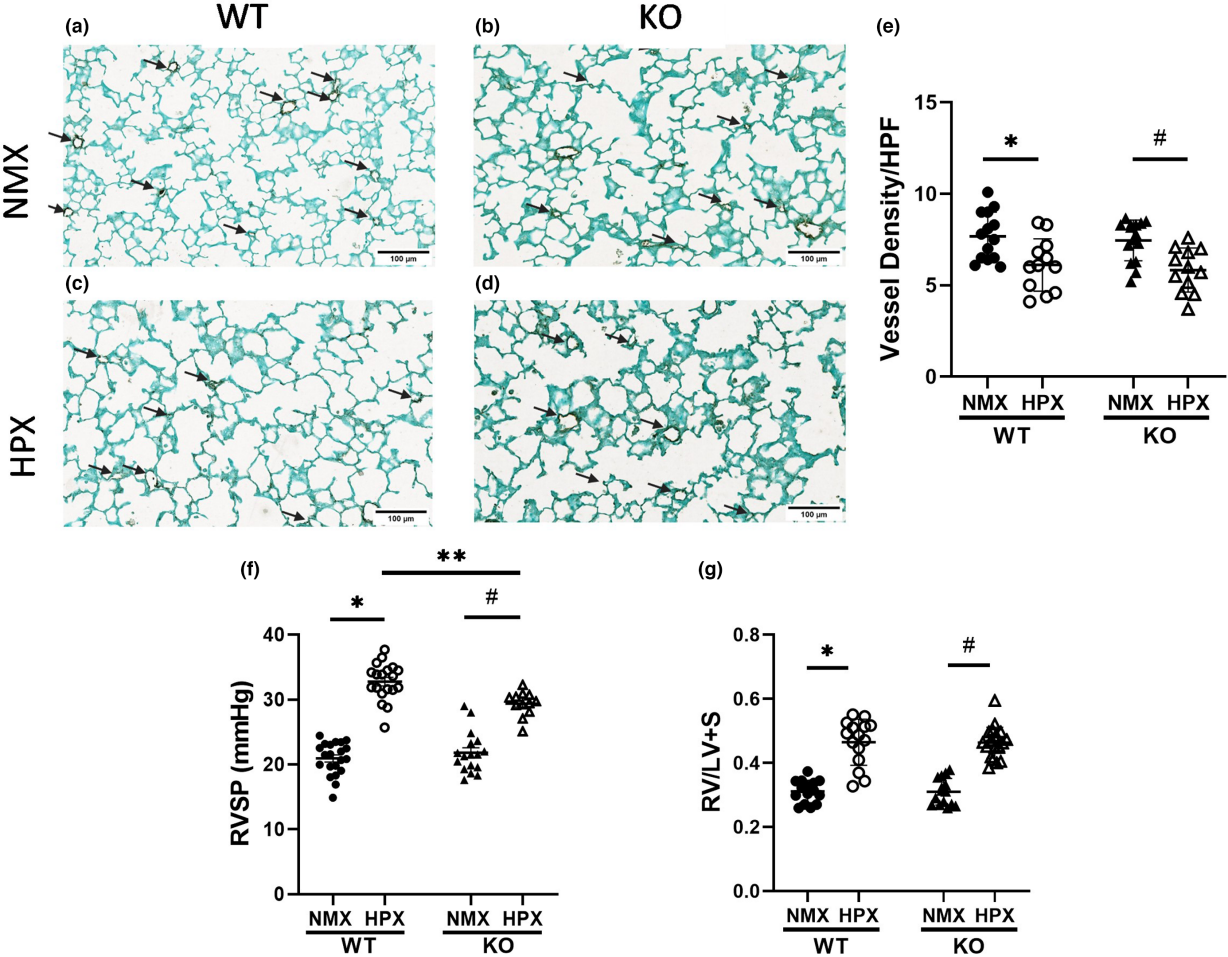
*Tph1* KO mice display comparable hypoxia‐induced decrease in vessel density and RVH when compared with WT mice. (a–d) Representative images of von Willebrand Factor (vWF; purple) stained lung sections from 2‐week‐old WT and *tph1* KO mice in normoxia and hypoxia. Arrows indicate vessels <30 μm, scale bar = 100 μm. (e) Vessel density in WT and *tph1* KO mice in normoxia and hypoxia, *p < 0.01 and ^#^ *p* < 0.01 by two‐way ANOVA, *n* = 14 WT NMX (7M, 7F), *n* = 15 KO NMX (8M, 7F), *n* = 12 WT HPX and KO HPX (7M, 5F), ns for sex. (f) RVSP in WT and TPH1 KO mice in normoxia and hypoxia, *p < 0.0001, **p < 0.01, and ^#^ *p* < 0.0001 by two‐way ANOVA, *n* = 19 WT NMX (11M, 8F), *n* = 17 KO NMX (8M, 9F), *n* = 19 WT HPX (11M, 8F), *n* = 12 KO HPX (6M, 6F), ns for sex. (g) RV/LV + S weights in WT and KO mice in normoxia and hypoxia, *p < 0.0001 and ^#^ *p* < 0.0001 by two‐way ANOVA, *n* = 15 WT NMX (9M, 6F), *n* = 15 KO NMX (8M, 7F), *n* = 15 WT HPX (9M, 6F), *n* = 20 KO HPX (11M, 9F), ns for sex. HPF, high powered field; HPX, hypoxia; KO, knock‐out; NMX, normoxia; RV/LV + S, right ventricle divided by left ventricle plus septum; RVSP, right ventricular systolic pressure; WT, wild‐type.

*Tph1* KO mice display comparable hypoxia‐induced alveolar simplification to WT mice, with similar decreased alveolar SA and increased MLI (Figure 4a–f). SA decreased 18% in hypoxia‐exposed KO mice compared with a 14% decrease in WT mice (Figure 4e, *p < 0.0001, ^#^ *p* < 0.0001). Hypoxia exposed KO mice display a 15% increase in MLI compared with a 14% increase in WT mice (Figure 4f, *p < 0.0001, ^#^ *p* < 0.0001). Neonatal *tph1* KO mice and WT mice have similar decreased density of small pulmonary arteries following hypoxia (Figure 5a–e). The density of vWF stained vessels <30 μm decreased 28% in *tph1* KO mice and 26% in WT mice with hypoxia exposure (Figure 5e, *p < 0.01, ^#^ *p* < 0.01).

### 3.4 Hypoxia‐induced increase in RVSP is attenuated in *tph1* KO mice compared with WT mice; however, hypoxia‐induced RVH is comparable between KO and WT mice

Prior studies in our lab have shown that pharmacologic inhibition of 5‐HT signaling via the 2A receptor prevents PH in neonatal mice (decreased RVSP and RVH) treated with bleomycin (Delaney et al., 2018). In the present study, we assessed whether *tph1* deletion, resulting in decreased peripheral 5‐HT, protects against hypoxia‐induced PH. We found that *tph1* KO mice display attenuation of hypoxia‐induced increase in RVSP compared with WT mice; however, RVH was not different between KO and WT mice. RVSP increased 26% in *tph1* KO mice compared with a 36% increase in WT mice (Figure 5f, *p < 0.0001, ^#^ *p* < 0.0001, **p < 0.01). Hypoxia‐exposed *tph1* KO and WT mice both display a 33% increase in RVH (Figure 5g, *p < 0.0001, ^#^ *p* < 0.0001).

### 3.5 Platelet and plasma 5‐HT is decreased in hypoxia‐exposed WT mice compared with WT normoxic mice

**FIGURE 6:**
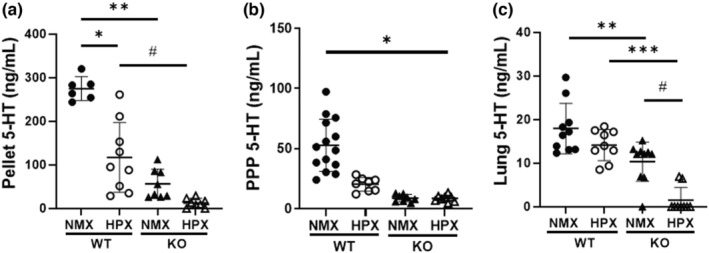
Plasma and platelet 5‐HT is decreased after 2 weeks of hypoxia exposure. (a) Platelet pellet 5‐HT levels of 2‐week‐old WT and KO mice in control conditions and hypoxia exposure, *p < 0.0001, **p < 0.0001, ^#^ *p* < 0.008 by two‐way ANOVA, *n* = 6 WT NMX (3M, 3F), *n* = 8 KO NMX (5M, 3F), *n* = 9 WT HPX (4M, 5F), *n* = 8 KO HPX (3M, 5F), ns by sex. (b) PPP 5‐HT levels of WT and KO mice in control conditions and with hypoxia exposure, *p < 0.0001 by one‐way ANOVA, *n* = 14 WT NMX (9M, 5F), *n* = 8 KO NMX (4M, 4F) mice *n* = 8 WT HPX (4M, 4F), *n* = 8 KO HPX (4M, 4F), ns by sex. (c) Lung 5‐HT levels of WT and KO mice in control conditions and with hypoxia exposure, **p < 0.0026, ***p < 0.0001, ^#^ *p* < 0.001 by two‐way ANOVA, *n* = 10 WT NMX (6M, 4F), *n* = 9 WT HPX (5M, 4F), *n* = 10 KO NMX (6M, 4F), *n* = 9 KO HPX (5M, 4F), ns by sex. HPX, hypoxia; KO, knock‐out; NMX, normoxia; PPP, platelet poor plasma; WT, wild‐type.

Based on our prior work demonstrating both increased plasma 5‐HT and pulmonary *tph1* expression, as well as accumulation of platelets, the primary source of 5‐HT within the lungs of neonatal mice with bleomycin‐induced PH, we hypothesized that circulating and pulmonary 5‐HT would be increased in neonatal mice following hypoxia exposure (Davizon‐Castillo et al., 2020). As the majority of circulating 5‐HT is stored within platelet dense granules, we measured both platelet and platelet poor plasma 5‐HT levels. Surprisingly, 5‐HT was decreased in both platelet and platelet poor plasma following 2 weeks of hypoxia in neonatal mice (Figure 6a, *p < 0.0001; Figure 6b, *p < 0.0001). Lung 5‐HT in WT normoxic and hypoxic mice was not different. Lung 5‐HT decreased 23% in hypoxia‐exposed KO mice when compared with lung 5‐HT of normoxic KO mice; of note, these values were low, close to the limit of detection of the assay (Figure 6c, ^#^ *p* < 0.001).

## 4 DISCUSSION

We previously reported that both pulmonary expression of TPH1, the rate limiting step in circulating 5‐HT synthesis, and plasma 5‐HT are increased in neonatal murine bleomycin‐induced PH and BPD (Delaney et al., 2018). Additionally, we reported that pharmacologic blockade of the 5‐HT 2A receptor prevents bleomycin‐induced pulmonary vascular remodeling and PH (Delaney et al., 2018). Based upon our prior work and studies in adult mice demonstrating that genetic deletion of tph1 protects against the development of PH, we hypothesized that hypoxia would result in increased lung and circulating 5‐HT in WT mice and genetic deletion of *tph1* would ameliorate hypoxia‐induced alveolar simplification, reduction in vessel density, and PH in neonatal mice. We tested this hypothesis in WT and *tph1* KO neonatal mice that either remained at Denver altitude or were exposed to hypobaric hypoxia for 2 weeks. We report that *tph1* KO mice display similar alveolar development, pulmonary vessel density, and pulmonary pressures to WT mice at baseline. Surprisingly, we found that circulating (plasma and platelet) 5‐HT decreased following hypoxia exposure and while *tph1* KO neonatal mice exhibited mild attenuation of hypoxia‐induced increase in RVSPs, *tph1* KO neonatal mice were not protected against hypoxia‐induced alveolar simplification, reduction in pulmonary vessel density, or RVH.

Our first major finding is that alveolar development, pulmonary vascular density, RVSPs and right heart size are similar between *tph1* KO and WT mice at baseline. 5‐HT is detected in fetal human lungs as early as 8 weeks gestation and increases lung fluid absorption in fetal guinea pigs close to term (Chua & Perks, 1999; Cutz et al., 1985; Pan et al., 2006). However, to our knowledge there are no studies evaluating 5‐HT's impact on fetal lung alveologenesis or angiogenesis. We report that peripheral 5‐HT depletion does not disrupt the parameters of alveolar development and vascular development measured at the time point of our study. It is possible that 5‐HT is not a critical mitogen or growth factor required for normal murine lung development or that *tph1* KO mice have sufficient levels of 5‐HT required for normal lung development as *tph1* KO mice synthesize peripheral 5‐HT via *tph2* within enteric neurons (Walther et al., 2003). We found that RVSPs in 2‐week‐old 5‐HT deficient mice (*tph1* KO) are similar to WT mice. Our findings in 2‐week‐old mice agree with findings in adult *tph1* KO mice (Le Cras et al., 2002). Deletion of the 5‐HT 2B receptor gene leads to embryonic and neonatal death attributed to cardiac defects (Nebigil et al., 2000). While we found that heart size did not differ between KO and WT mice, our cardiac evaluation of *tph1* KO mice was limited to RV and LV mass. As such, we cannot draw additional conclusions regarding whether 5‐HT deficiency affects cardiac development or function.

Exogenously administered 5‐HT potentiates the development of PH in rats (Eddahibi et al., 1997). Decreased platelet storage of 5‐HT, resulting in elevated plasma 5‐HT, leads to the development of PH in patients with platelet storage disease and fawn hooded rats (Herve et al., 1990; Le Cras et al., 1999). However, reports are conflicting regarding whether circulating 5‐HT is increased in adults with PH or rodents with experimental PH (Herve et al., 1990, 1995; Kereveur et al., 2000; Lederer et al., 2008; Zeinali et al., 2014). Similar to prior reports in adult hypoxic mice, we found that both platelet and plasma 5‐HT are decreased in neonatal mice with hypoxia‐induced PH (Abid et al., 2012). As lung 5‐HT levels do not change and both plasma and platelet 5‐HT decrease following hypoxia, we speculate that hypoxia results in decreased peripheral synthesis of 5‐HT. Our findings in hypoxic 2‐week‐old mice differ from our prior findings in bleomycin‐treated neonatal mice where we found an increase in plasma 5‐HT (Delaney et al., 2018). While the mechanisms for these differences are unclear, bleomycin causes transient thrombocytopenia and induces endothelial injury, potentially increasing plasma 5‐HT via platelet activation and subsequent release of 5‐HT (Hilgard & Hossfeld, 1978). The results of our studies, in which we utilize two different models of neonatal PH, highlight the importance of evaluating disease processes at various time points and in multiple models to broaden our understanding of complex interactions that underlie disease etiology.

TPH1 pharmacologic inhibition attenuates PH in several adult rodent models (Abid et al., 2012; Aiello et al., 2017; Ciuclan et al., 2013). Blockade of 5‐HT via inhibition of the 2A receptor decreases fetal pulmonary vascular resistance in the ovine fetus with PH and prevents the development of PH and pulmonary vascular remodeling in a neonatal murine model of bleomycin‐induced BPD and PH (Delaney et al., 2013, 2018). Based on these findings, we hypothesized that hypoxia‐induced PH would be prevented in *tph1* KO neonatal mice. While we found that hypoxia‐induced increase in RVSP was attenuated in *tph1* KO mice compared with WT mice, *tph1* KO mice were not protected against hypoxia‐induced RVH. The reasons for lack of protection against PH is unclear, but not surprising, as 5‐HT did not increase in this model. Additionally, we did not measure pulmonary 5‐HT transporter or receptor expression, it is feasible that changes in receptor expression may also drive the lack of protection.

There are a few potential limitations to our study. Alveolar development, pulmonary vascular development, and pulmonary pressures are dynamic measures, and our study evaluated a single time point during the alveolar stage of lung development. This raises the question of whether assessment at an earlier timepoint would reveal baseline differences in alveolar development or vascular density in *tph1* KO mice compared with WT mice. We did not perform pulmonary function studies to assess whether similar alveolar development between WT and *tph1* KO mice correlated with lung function as determined by lung compliance and airway reactivity. Furthermore, we measured 5‐HT levels in the circulation and lung at the end of our study. Whether these findings reflect levels across the 2‐week study period is unknown. Additionally, 5‐HT modulates systemic blood pressure, a value we did not collect in the present study (Watts et al., 2012). Systemic hypotension may falsely lower RVSP values and impact data analysis. A consideration for future studies is to obtain neonatal echocardiograms to assess cardiac output.

We conclude that *tph1* KO neonatal mice are not protected against hypoxia‐induced alveolar simplification, reduction in pulmonary vessel density, or RVH. While genetic and pharmacologic inhibition of tph1 has protective effects in adult models of PH, our results suggest that tph1 inhibition may have some utility in decreasing pulmonary vasoconstriction but would not be beneficial in treating PH in neonates with PH associated with BPD.

## AUTHOR CONTRIBUTIONS

Danielle S. Roberts, Laura G. Sherlock, Janelle N. Posey, Jamie L. Archambault, Eva S. Nozik, and Cassidy A. Delaney made substantial contributions to conception and design, acquisition of data, or analysis and interpretation of data:. Danielle S. Roberts, Laura G. Sherlock, Janelle N. Posey, Eva S. Nozik, and Cassidy A. Delaney drafted the article or revised it critically for important intellectual content. Danielle S. Roberts and Cassidy A. Delaney final approval of the version to be published.

## FUNDING INFORMATION

Financial support was provided by National Heart, Lung, and Blood Institute Grants K08 HL132041‐01 (to C.D.), and 1R35HL139726–01 (to E.S.N.).

## CONFLICT OF INTEREST

The authors have no conflict of interest or disclosures.

## INFORMED CONSENT

No patient consent was required for this manuscript.
